# Genomic and metabolic comparison with *Dickeya dadantii* 3937 reveals the emerging *Dickeya solani* potato pathogen to display distinctive metabolic activities and T5SS/T6SS-related toxin repertoire

**DOI:** 10.1186/1471-2164-15-283

**Published:** 2014-04-15

**Authors:** Jacques Pédron, Samuel Mondy, Yannick Raoul des Essarts, Frédérique Van Gijsegem, Denis Faure

**Affiliations:** 1 UPMC Univ Paris 06, UMR 1392, IEES Paris (Institute of Ecology and Environmental Sciences), 46 rue d’Ulm, F-75005 Paris, France; 2 INRA, UMR 1392, IEES Paris (Institute of Ecology and Environmental Sciences), 46 rue d’Ulm, F-75005 Paris, France; 3 CNRS, UPR2355, ISV (Institut des sciences du végétal), Avenue de la terrasse, F-91198, Gif-sur-Yvette, France; 4 FN3PT/RD3PT, Fédération Nationale des Producteurs de Plants de Pomme de Terre, 43-45 Rue de Naples, F-75008 Paris, France

**Keywords:** PKS, NRPS, CDI, Rhs, Dickeya dadantii, Dickeya solani, Plant pathogen interactions

## Abstract

**Background:**

The pectinolytic enterobacteria of the *Pectobacterium* and *Dickeya* genera are causative agents of maceration-associated diseases affecting a wide variety of crops and ornamentals. For the past decade, the emergence of a novel species *D. solani* was observed in potato fields in Europe and the Mediterranean basin. The purpose of this study is to search by comparative genomics the genetic traits that could be distinctive to other *Dickeya* species and be involved in *D. solani* adaptation to the potato plant host.

**Results:**

*D. solani* 3337 exhibits a 4.9 Mb circular genome that is characterized by a low content in mobile elements with the identification of only two full length insertion sequences. A genomic comparison with the deeply-annotated model *D. dadantii* 3937 strain was performed. While a large majority of *Dickeya* virulence genes are shared by both strains, a few hundreds genes of *D. solani* 3337, mostly regrouped in 25 genomic regions, are distinctive to *D. dadantii* 3937. These genomic regions are present in the other available draft genomes of *D. solani* strains and interestingly some of them were not found in the sequenced genomes of the other *Dickeya* species. These genomic regions regroup metabolic genes and are often accompanied by genes involved in transport systems. A metabolic analysis correlated some metabolic genes with distinctive functional traits of both *D. solani* 3337 and *D. dadantii* 3937. Three identified *D. solani* genomic regions also regroup NRPS/PKS encoding genes. In addition, *D. solani* encodes a distinctive arsenal of T5SS and T6SS-related toxin-antitoxin systems. These genes may contribute to bacteria-bacteria interactions and to the fitness of *D. solani* to the plant environment.

**Conclusions:**

This study highlights the genomic specific traits of the emerging pathogen *D. solani* and will provide the basis for studying those that are involved in the successful adaptation of this emerging pathogen to the potato plant host.

## Background

The pectinolytic enterobacteria of the *Pectobacterium* and *Dickeya* genera are causative agents of maceration-associated diseases affecting a wide variety of crops and ornamentals [1]. During the past century, the species *Pectobacterium atrosepticum* and *Pectobacterium carotovorum* dominated the pathogenic populations in samples collected from black-leg and soft-rot symptoms of potato plants and tubers under temperate climates. Since the 1970’s, *Dickeya* strains, mostly *D. dianthicola*, were also detected in potato fields in Europe. More recently, in the 2000’s, a novel species called *Dickeya solani*[2,3], emerged among the pectinolytic enterobacteria recovered from potato symptoms in several countries. The emergence and challenges of the *Dickeya* species in potato production have been deeply discussed by Toth *et al.*[2].

Genetic markers and biochemical tests allowed to clearly distinguishing these novel pathogenic strains from the established *Dickeya* species, including *D. dianthicola*, *D. dadantii*, *D. zeae*, *D. chrysanthemi*, *D. paradisiaca* and *D. dieffenbachia*[4-7]. Aside the current effort on the taxonomic description of *D. solani*, questions arose about the origin, adaptation, ecology and, in an applied issue, the control of this emerging plant pathogen on potato cultures. Even if only a few genetic markers were analysed in several *D. solani* isolates from different countries, their high similarities suggested a clonal origin of *D. solani* populations affecting the potato plant host [4-6,8]. Remarkably, under greenhouse conditions at a high temperature (28°C), when the two bacterial species *D. solani* and *D. dianthicola* are coinoculated, *D. solani* isolates outcompete those of the other *Dickeya* species, revealing a high efficiency for colonizing potato roots and stems [9,10]. The genes and functions involved in these traits are still unknown.

For several decades, *D. dadantii* strain 3937 was widely used as a model system for research on the molecular biology and pathogenicity of pectinolytic enterobacteria. The *D. dadantii* 3937 strain remains, until today, the *Dickeya* isolate in which virulence factors and host-interacting functions are the most studied [1,11]. Its annotated genome is available [12], hence is used for fruitful comparison with the released draft and completed genomes belonging to *Dickeya* and other plant pathogenic genera.

The *D. solani* strain 3337 (also named RNS 08.23.3.1A) has been isolated from potato in France in 2008 [13] and its aggressiveness on potato plants has been confirmed [14]. In this paper, we reported a genomic and metabolic comparison of the *D. solani* strain 3337 and *D. dadantii* strain 3937.

## Methods

### Bacterial strains, culture conditions and metabolic assays

*D. solani* 3337 (=RNS 08.23.3.1A) [13] and *D. dadantii* 3937 [12] were routinely cultured in TY medium (tryptone 5 g/L, yeast extract 3 g/L, agar 1.5%) at 30°C. The metabolic abilities of *D. solani* 3337 and *D. dadantii* 3937 were investigated using the carbon source microplates PM1 and PM2A and the nitrogen source microplate PM3B (Biolog, France). Sodium pyruvate was used as a carbon source for testing the nitrogen sources. The experiment was duplicated. According manufacturer’s instructions, OD_590nm_ (Tecan spectrophotometer) was read at the inoculation time (t_i_) and after a 48 h incubation (t_f_); variation ΔOD = t_f_-t_i_ was calculated for each of the tested nitrogen and carbon sources. A ΔODvalue < 0.25 indicated that bacteria did not metabolize the nutrients; 0.25 ≤ ΔOD < 1 revealed a weak metabolic activity and ΔOD ≥ 1 referred to a very efficient metabolic activity.

### Genome sequencing of *D. solani* 3337

Two DNA-libraries were constructed by Eurofins Genomics (France) using the TruSeq(TM) SBS v3 sequencing kit: a shotgun (SG) paired-end library with a fragment size between 150 to 500 bp and a long jumping distance (LJD) mate-pair library with an insert-size average of 5.7 kbp. The two libraries were sequenced using 2×100 bp paired-end read module of IlluminaHiSeq 2000 by Eurofins Genomics (France). Reads were trimmed on quality and length. Sequence assembly was carried out using the CLC Genomics Workbench v5 (CLC bio, Aarhus, Denmark) with a read length of 0.5 and a similarity of 0.8. Forty-two contigs were obtained with a length ranging from 2.1 to 483 kbp. The scaffolding was processed using SSPACE basic v2.0 [15].

#### *In silico finishing*

The *in silico* finishing of some gaps was carried out by mapping (read length of 0.9 and similarity of 0.95) the mate-pair reads on each of the 5 kbp contig-ends since the *de novo* assembly software faced difficulty during assembly in repeated regions. We used the borders of the gap regions as anchor and retrieved the reads in both orientations in order to perform a new *de novo* assembly on these regions. The mapped reads were collected and both orientation R1 and R2 were retrieved. The reads were used for *de novo* local assembling (read length of 0.5 and similarity of 0.8). Additional gaps were closed by Sanger sequencing of PCR amplicons.

### Annotation of the *D. solani* 3337 genome and comparison with *D. dadantii* 3937

The functional annotation of predicted genes of *D. solani* 3337 was achieved using the RAST server [16] with the Glimmer 3 gene caller [17]. As the genome annotation of *D. dadantii* 3937 was performed in 2004 with the version 2 of Glimmer, this genome was also re-annotated with the same RAST server in order to compare genes predicted with the same algorithm. In *D. solani* 3337, the remaining gaps affect 15 genes that were not considered in the subsequent analysis.

Synteny analysis was performed by using the MAUVE software [18]. Average nucleotide identity (ANI blast) was computed using the Jspecies package [19]. Genome to genome comparison was performed by bi-directional protein-protein BLAST sequence comparison of translated open reading frames (ORFs) with a 10^-5^ e-value threshold. Genes of both species were considered as strain-specific if identity of the encoded protein was lower than 80% of full-length amino acids sequence. For comparison, average identity level of the proteins encoded by conserved genes, calculated as the mean of all identity percentages, is 96%. If local alignments were too short as regard to the length of similar sequences, we performed a nucleotide BLAST on full-length DNA sequences with similar thresholds (10^-5^ e-value, 80% identity of full length sequence). This allowed us to eliminate false strain-specific genes. In some cases, identities were lower than 80% due to differences in predicted ORF length. Such differences in length of similar sequences was the consequence of wrong prediction of start codons or when two predicted ORFs in one species matched with one ORF in the other one. All similar but strain-specific sequences that differ by more than 10% in length were manually analysed to detect putative wrong start codon prediction or occurrence of stop codons that could explain the interruption of one ORF.

### Comparison of the *D. solani* 3337 genome with other *Dickeya* draft genomes

The *D. dadantii* 3937 genome resulted from Sanger genomics. The production of reads mimicking Illumina reads of the *D. dadantii* 3937 genome was performed using Metasim [20]. A set of 2,000,000 reads derived from the *D. dadantii* 3937 genome with an average size of 100 nucleotides were produced and used for mapping approaches as described below.

The Illumina reads of *D. solani* 3337 and simulated reads from *D. dadantii* 3937 were mapped using the draft genomes of the four *D. solani* strains IPO2, MK10, MK16 and LMG25865 [21] and the *D. dadantii* genome and draft genomes of the strains *D. dadantii* 3937, NCPPB3537 and NCPPB898. Two mappings were performed at high (0.95 of identity on 0.9 of read length) and mild stringency threshold (0.8 of identity on 0.5 of read length). Using such *in silico* DNA-DNA hybridizations, a high proportion of mapped reads will indicate a strong identity between genome sequences. Then, the mapping files constructed using high stringency parameters were processed for SNP analysis.

In addition, the presence of genomic clusters in the *D. solani* and *D. dadantii* species was searched with a nucleotide BLAST analysis using the sequences of the specific clusters as queries against a database constituted by the *D. solani* and *D. dadantii* genomes (e-value threshold = 10^-50^).

The search for the presence of *D. solani*-specific and D. dadantii 3937 specific-genes in other *Dickeya* species was performed by bi-directional protein-protein BLAST sequence comparison (e-value threshold 10^-5^) with the *D. paradisiaca* 703 strain (formerly named *D. dadantii* 703, GenBank accession number CP001654), the *D. zeae* 586 strain (formerly named *D. dadantii* GCF_000025065.1) and *the D. chrysanthemi* 1591 strain (formerly named *D. zeae* CP001655) as reclassified by Pritchard *et al.*[21].

### Genome accession numbers

The *D. solani* 3337 genome sequence has been deposited at DDBJ/EMBL/GenBank under the accession n°AMYI00000000. Sequence data used in this article can be found in the GenBank/EMBL data libraries under the following accession numbers: *D. solani* strains IPO2222 (CM001859), MK10 (CM001859), MK16 (CM001842), LMG25865 (CM001860), and *D. dadantii* strains 3937 (CP002038), NCPPB3537 (CM001982), NCPPB898 (CM001976).

## Results and discussion

### General features of the *D. solani* 3337 genome

The published sequence of *D. solani* 3337 is composed of eleven contigs (from 4.5 kbp to 1.11 Mbp) grouped in a single scaffold with 1,075 coverage in average. The *in silico* approach permits to close 32 gaps and to map the 7 *rrs* regions. Hence, the *D. solani* 3337 genome consists of a unique 4.9 Mb circular chromosome with a 56.1% GC content and contains no plasmid. These features are in accordance with data of the *Dickeya* genome and draft genomes that are available in public databases [7,21]. Using the RAST server with the Glimmer 3 caller, gene prediction and automatic annotation predicted 4530 protein-coding sequences, 72 tRNA genes and 7 rRNA regions.

### Comparative genomics between *D. solani* 3337 and *D. dadantii* 3937: an overview

To go further into the *D. solani* 3337 genome analysis, we took advantage of the manually annotated genome of the model *D. dadantii* 3937 strain [12]. To allow a precise comparison, a novel gene prediction with Glimmer 3 was achieved on the *D. dadantii* 3937 genome leading to the prediction of 4740 protein-coding sequences and 97 RNA genes. Noticeably, 526 new genes were identified with the Glimmer 3 software as compared to the previous gene prediction but only 27 have a known or predicted function. All the other CDS are annotated as hypothetical and 85% (448) are smaller than 60 amino acids.

**Figure 1:**
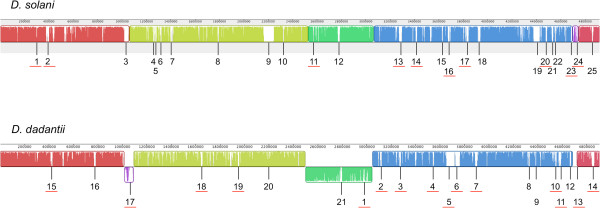
**Synteny between *D. solani *3337 and *D. dadantii *3937 genomes.** Synteny analysis was performed using the MAUVE software. The two big genomic islands located in different positions in both genomes are drawn in purple (*D. solani*-GR24, *D. dadantii*-GR17). Numbers indicated the positions of the genomic regions containing strain-specific genes. The genomic regions carrying indication of horizontal transfer are underlined in red.

The relatedness between these genomes was first analysed using the MAUVE program (Figure 1). This reveals a very high synteny between both genomes that was only interrupted by a big inversion between two *rrs* ribosomal RNA-encoding operons present in opposite orientation, and by the insertion at different positions in both genomes of a region of 48.4 kb and 74.5 kb respectively. These regions contain both common and strain-specific genes (see below).

Genes conserved in both strains were identified by performing bidirectional best hit analysis at the protein level and retaining only the genes that encode proteins exhibiting at least 80% identity on full length amino acid sequence. Manual check of remaining genes allowed the identification of additional conserved genes excluded from the previous test because of prediction or alignment ambiguities (see Methods for details). About 3700 genes have homologues in both strains. This corresponds to 78-82% of the total protein-coding sequences. This is in fact comparable to the core genome size of certain bacterial species such as *Porphyromonas gingivalis* or *Streptococcus pneumoniae*[22,23] and is considerably larger than that of other enterobacterial species such as *E. coli*[24]. The closeness of both strains is supported by the 94% average nucleotide identity (ANI) value, an analysis that has emerged as one of the predominant genomics alternatives to DNA-DNA hybridization and stated that two strains belong to the same species if their pairwise ANI values are higher than 96% [19]. It is also in accordance with previous taxonomic studies that place *D. solani* and *D. dadantii* species close to each other [21,6].

### Most virulence genes are conserved between *D. solani* 3337 and *D. dadantii* 3937

#### The main virulence factors

The main virulence determinants in soft rot enterobacteria are plant cell wall degrading enzymes (PCWDEs) that cause extensive tissue maceration during the latter stages of infection [1,11]. The *D. dadantii* genome contains 23 pectinases encoding genes including pectate and pectin lyases, polygalacturonases, pectin-methyl and acetyl esterases. All corresponding genes are present in *D. solani* except the pectin lyase encoding *pnlH* gene (see below) and the *pehK* gene (locus tag Dda3937_00206) encoding a predicted polygalacturonase that is present in all 4 *Dickeya* for which complete genomes are available, several *Pectobacterium* strains and several other enterobacteria. Both strains shared the same battery of cellulases and extracellular proteases as well as iron uptake systems, production of antioxydants like indigoidine and systems involved in defence against the plant oxidative burst or antimicrobial peptides [25-27,1,11]. Concerning protection to osmotic stress however, the *ousA* gene encoding the major osmoprotectant uptake system in *D. dadantii* 3937 is missing in *D. solani*. Interestingly, an *ousA* disruption in *D. dadantii* 3937 highly enhanced bacterial virulence on potato tubers and enhanced Pel production *in vitro* under micro-aerobiosis conditions. This might be correlated to the observation that the less NaCl-tolerant *Dickeya* strains are the more virulent in potato tubers [28].

#### *Proteins secretion systems of type 1 to 6 and toxin/antitoxin systems*

**Figure 2:**
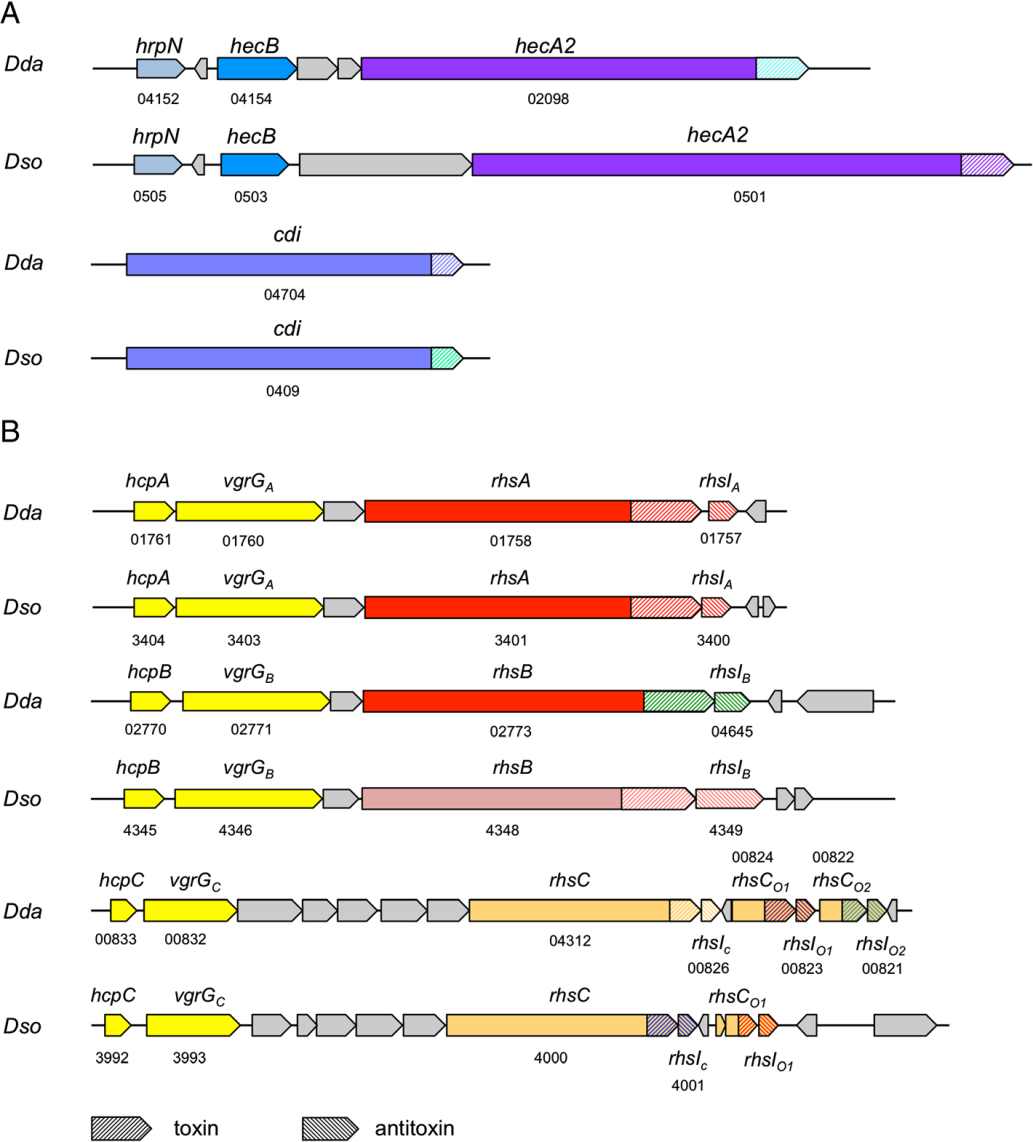
**Repertoire of T5SS and T6SS-related toxin/antitoxin systems in *D. dadantii *3937 and *D. solani *3337.** Comparison of *D. dadantii* (*Dda*) and *D. solani* (*Dso*) T5SS-related *cdi***(A)** and *rhs* loci **(B)**. Homologous gene domains are depicted in the same colour. T6SS *hcp* and *vgrG* genes are depicted in yellow. Toxin CT domains are depicted in right-leaning streaks and corresponding antitoxin genes are depicted in similarly coloured left-leaning streaks. Locus Tag numbers are indicated for *D. dadantii* genes and Glimmer 3 IDs for *D. solani* genes.

Like *D. dadantii*[1], *D. solani* possesses all six known gram-negative protein secretion systems and the corresponding genes are highly conserved in both strains. The Stt second type 2 secretion system (T2SS) present in the *D. dadantii* 3937 strain is however missing in *D. solani* 3337 as well as the PnlH pectin lyase that is presented to the outer side of the outer membrane by this Stt system [29]. While proteins shown or predicted to be secreted by secretion systems 1 to 3 are conserved in both strains, the repertoire of proteins transiting to T5SS and T6SS is more variable. Interestingly, these two systems have been shown to be involved in toxin delivery. T5SS encompass two-partner secretion systems called Tps that consist of an outer membrane TspB protein allowing the secretion of a large TspA multidomain protein composed of an N-terminal transport domain, a large hemagglutinin-like region that is proposed to form a fiber-like structure and, in some cases, a C-terminal toxin domain. *D. dadantii* 3937 possesses two Tps systems that have been shown to act in contact-dependent growth inhibition (CDI) by delivering the C-terminal toxin domain of TspA/CdiA (CdiA-CT) to target cells. The two *D. dadantii* 3937 CdiA-CT toxins differ, the first one being a tRNase and the second one harboring DNase activity. Each Cdi system also encodes a specific CdiI immunity protein that interacts with the cognate CdiA-CT toxin to prevent auto-inhibition [30,31]. In *D. solani* 3337, two Cdi systems are also present at similar locations than in *D. dadantii* 3937. They share a high similarity with the corresponding *D. dadantii* 3937 proteins except for the 200 C-terminal bp of CdiA corresponding to the toxin CT part (Figure 2A). *D. dadantii* 3937 also possesses 3 copies of the toxin/antitoxin Rhs (Rearrangement HotSpot) system and two of them (RhsA and RhsB) were shown to mediate intercellular competition and harbor DNAse activity [31,32]. The Rhs proteins are large composite proteins consisting of a large N-domain containing YD-peptide repeats and a highly variable C-domain harboring toxic activity. The *rhs* encoding genes are followed by a small *rhsI* gene encoding an immunity protein that blocks the toxic activity of the corresponding Rhs-CT. *D. dadantii* 3937 possesses 3 *rhs* genes linked to hemolysin-coregulated protein (Hcp) and valine-glycine repeat protein G (VgrG) that encode the components of the external part of T6SS [33]. VgrG is required for *D. dadantii* 3937 Rhs inhibitor cell function pointing to the export of Rhs protein via a type 6 secretion system [31]. *D. solani* also carries 3 Rhs proteins located in the similar genomic regions as in *D. dadantii* 3937. The relatedness between *D. dadantii* and *D. solani* Rhs systems is variable: the RhsA system is totally conserved between both strains (91% identity) while RhsB proteins only show moderate similarity between both strains both in their N-domain and Rhs-CT domains. The RhsC locus encodes similar large N-domains but differs in the toxin/antitoxin moiety and also in the number and sequence of additional orphan Rhs-CT/RhsI pairs (Figure 2B). The two T5SS-related *cdi* systems are present in the other sequenced *Dickeya* species but only the *D. chrysanthemi* 1591 *cdi* N-end part is conserved with *D. solani* 3337, all other toxin Cterm are divergent. The 3 Rhs loci are present in *D. chrysanthemi* 1591 and *D. zeae* 586. Only RhsCDze_586_ is totally homologous to RhsCDso_3337_, all other Rhs proteins harbor divergent Cterm toxin motives. Interestingly, it should be noted that a T6SS system is missing in *D. paradisiaca* 703 strain (Additional file 1: Table S1).

#### *Production of plant hormones*

Among genes that may be involved in plant-bacteria interactions, it should be noted that the *D. dadantii iaaMH* genes (Dda3937_01279 and 01280) allowing the production of an auxin plant hormone, do not have counterparts in *D. solani*. Accordingly, *D. solani* produces only minute amounts of indolic compounds related to auxins as compared to *D. dadantii*[34].

#### *Virulence regulatory pathways*

In *D. dadantii*, a complex regulatory network composed of several global regulators [11] out of whom the GacAS two-component systems, the PecS and MfbR members of the MarR family of regulators, or the recently discovered new quorum-sensing system Vfm, have been shown to control virulence gene expression *in planta*[35-38]. All these regulators are present in *D. solani*. Out of the 17 members of the MarR family present in *D. dadantii* 3937 however, 3 corresponding genes (Dda3937_01219, 01245 and 03163; MfhR, MfiR and MfeR) are missing in *D. solani* 3337. These regulators were shown to be dispensable for full virulence expression in *D. dadantii* but they might have a role in other parts of the bacterial life cycle since in different bacteria, regulators of this family are involved in the sensing of signalling molecules [37]. By contrast, all *D. dadantii* regulators of the LacI family are present in *D. solani*. This family of regulators are very often related to global metabolism and some of them are involved in virulence in *D. dadantii* 3937 [39]. In addition, *D. solani* possesses functional *arcB* and *soxS* genes, two genes involved respectively in the aerobiosis/anaerobiosis switch and in oxidative defence regulation that are either mutated (*arcB*) or absent (*soxS*) in *D. dadantii* 3937 [40].

### Strain-specific genes in *D. solani* 3337 and *D. dadantii* 3937: an overview

A systematic analysis allowed the identification of 808 genes of *D. solani* 3337 and 1034 genes of *D. dadantii* 3937 that are present in one strain but not the other or that exhibit less than 80% identity (Table 1, Additional file 2: Tables S2 and S3). Among them, 506 *D. solani* 3337 and 617 *D. dadantii* 3937 genes encode hypothetical proteins having no related proteins in databases. It should be noted that 2/3 of them encode peptides of less than 60 amino acids for which transcription and translation is questionable. It should also be noted that only some thirty genes in each genome present sequence changes leading to the production of a truncated or out of phase protein (see Additional file 2: Tables S2 and S3), indicating that only a few strain-specific genes result from mutations in the corresponding gene in the other genome.

**Table 1:** **Overview of *D. solani *** **3337 and *D. dadantii *** **3937 specific encoding protein genes**

**Strain-specific genes**	***D. solani *** **3337**	***D. dadantii *** **3937**
**Total number**	**808**	**1034**
Hypothetical proteins (<60 aa)	506 (324)^1^	617 (403)^1^
Proteins with undefined function	52	75
Proteins with predicted function	250 (194)^2^	342 (183)^2^

^1^Numbers in brackets correspond to the number of proteins smaller than 60 amino acids.

^2^Numbers in brackets correspond to the number of strain-specific proteins that are also absent in other *Dickeya* species (*D. paradisiaca* 703, *D. zeae* 586 and *D. chrysanthemi* 1591).

**Figure 3:**
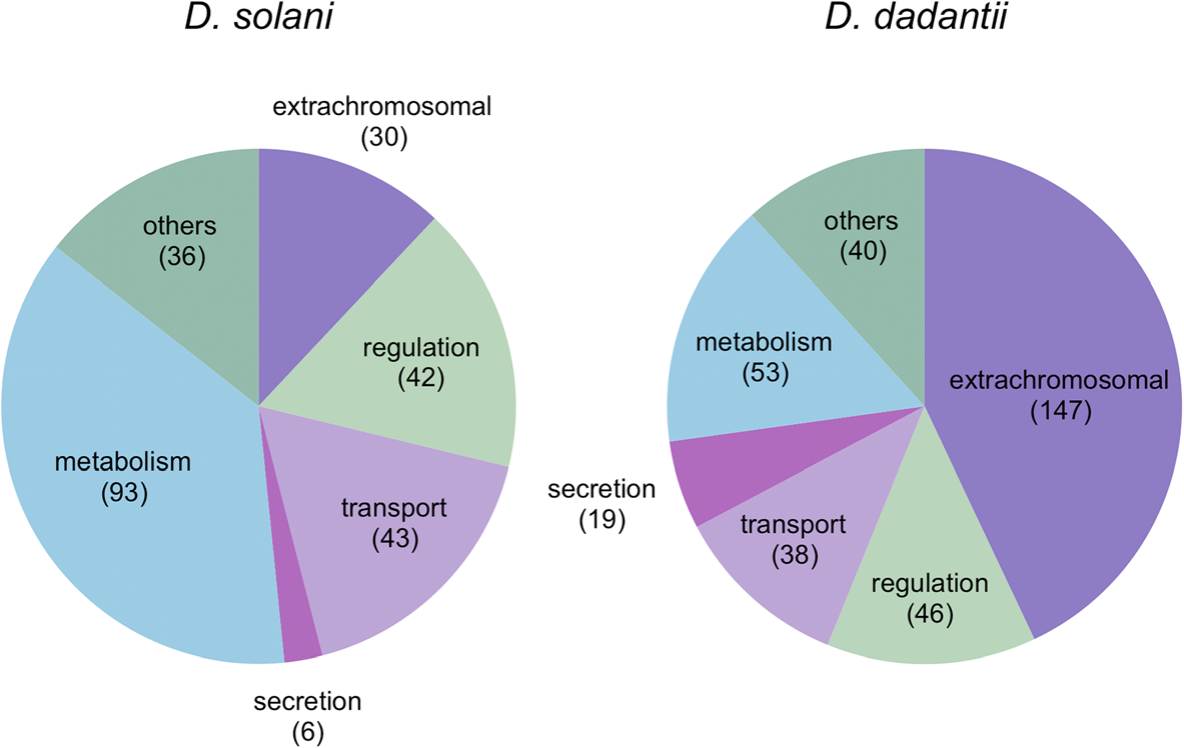
**Functional classification of *D. solani *3337 and *D. dadantii *3937 strain-specific genes.** The 342 (*D. dadantii* 3937) and 250 (*D. solani* 3337) strain-specific genes with known or predicted function were manually classified in functional categories. The number of genes in each category is indicated in brackets. Genes encoding hypothetical proteins and proteins of undefined function were excluded.

For 250 and 343 of these strain-specific genes respectively, a function is known or predicted, and for each strain about 200 genes are absent from other sequenced *Dickeya* species (Table 1, Additional file 2: Tables S2 and S3). All these strain-specific genes were manually classified in functional categories and the relative distribution of these categories was determined for both strains (Figure 3, Additional file 2: Tables S2 and S3). Though the number of specific genes involved in transport or regulation was roughly the same in both strains, the *D. dadantii* 3937 genome is clearly enriched in mobile elements and phage-associated genes while *D. solani* 3337 harbors more specific genes involved in metabolism. In both strains, more than 2/3 of strain-specific genes having a predicted function are clustered in genomic regions (GR) that are positioned along the chromosome (Figure 1).

#### *The D. solani 3337 genome is poor in mobile elements and phage-related genes and defective for CRISPR*

Insertion sequences (IS) and transposable elements may promote genome plasticity and contribute to bacterial adaptability [41]. While about 40% of the *D. dadantii 3937* strain-specific genes of known or predicted function (147 genes) are related to mobile elements or phages functions, only 30 such genes were identified in *D. solani*. This is due in part to the presence of a Tn4371 ICE (Integrative Conjugative Element) family element in GR5 (Additional file 2: Table S3) [42] and a complete prophage with morphogenesis genes related to *Haemophilus* HP1 and HP2 genes in *D. dadantii* 3937 (see Figure 1, Additional file 2: Table S3). Unlike *D. solani* 3337*, D. dadantii* 3937 also harbors a region with CRISPR (Clustered Regularly Interspaced Short Palindromic Repeats) sequences and associated genes (GR10, Additional file 2: Table S3). CRISPR are thought to provide acquired resistance to viruses in prokaryotes, as recently demonstrated in *Streptococcus thermophilus*[43]. Beside this, *D. dadantii* 3937 possesses two times more genes related to transposases (37 versus 15) than *D. solani* 3337. Accordingly, detection of IS elements using the ISBiotoul server (http://www-is.biotoul.fr) reveals the presence of only 2 full length IS (IS110 family: 3673879–3675 442; IS3/IS407 family: 2569337–2570527) in *D. solani* while 19 full length IS are present in *D. dadantii* 3937. Remarkably, the *D. solani* IS110-related sequence is inserted into a transposase gene in the GR16 region that contains in total 6 of the 15 *D. solani* transposase-related genes and a T6SS-related Hcp encoding gene (ID 3387). The other IS is surrounded by genes related to phage components in the GR11 region.

**Figure 4:**
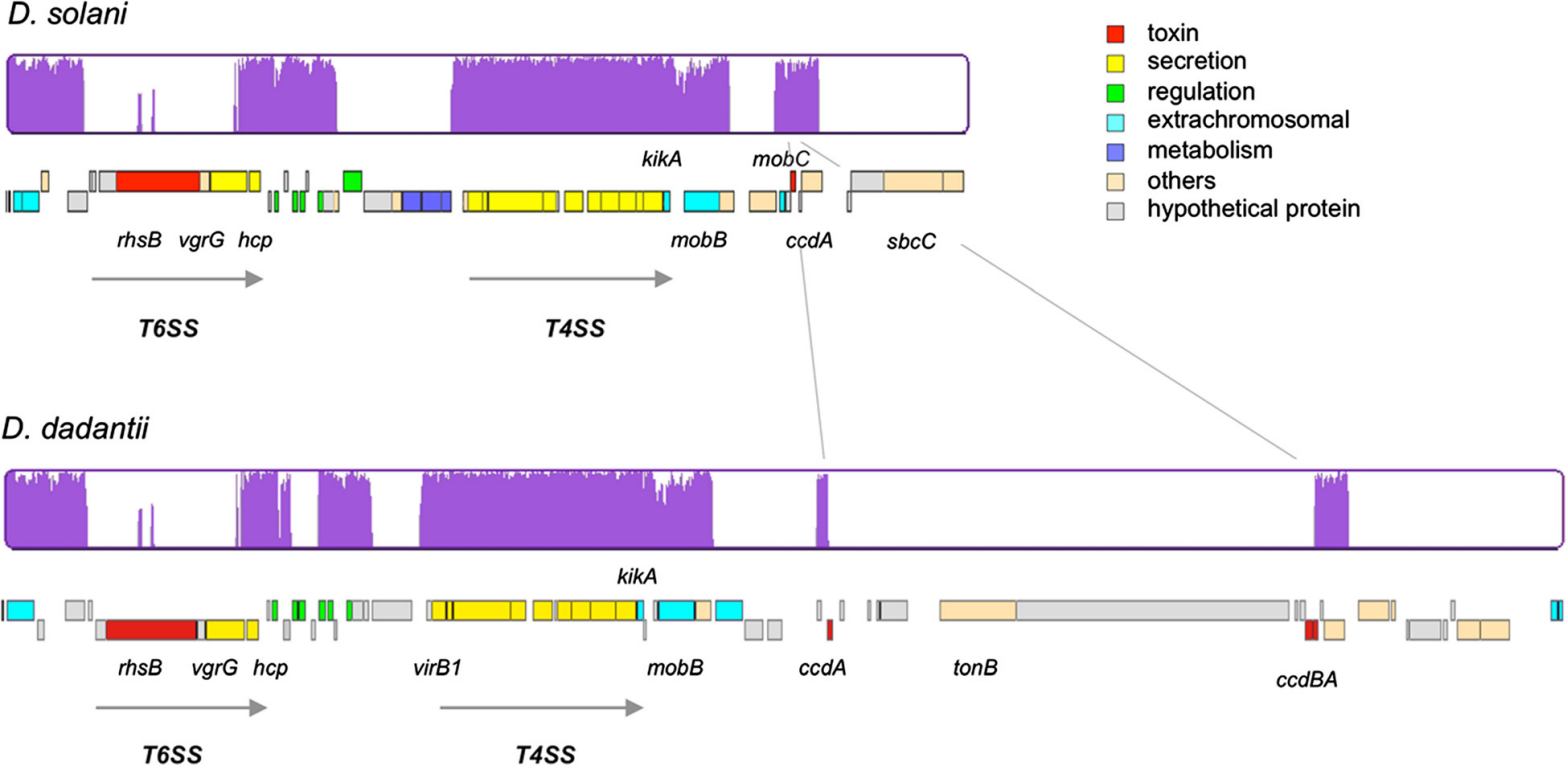
**Genetic organisation of the *D. solani *3337 and *D. dadantii *3937 genomic islands.***D. solani* 3337 genomic island (GR24, Figure 1 and Additional file 2: Table S2) encompass 51 genes (solani_g3_4305 to solani_g3_4355), *D. dadantii* 3937 genomic island (GR17, Figure 1 and Additional file 2: Table S3) encompasses 65 genes (3937_g3_3041 (locus tag Dda3937_00080) to 3937_g3_3105 (locus tag Dda3937_01864)). The purple diagrams represent the gene conservation in one region as compared to the other as calculated by the MAUVE software. The vertical lines link the conserved parts of the *D. solani* small region that is split by the insertion of additional genes in *D. dadantii*.

Most *D. dadantii* 3937-specific genomic regions (17 out of 22) contained transposase or integrase encoding genes that are indications of acquisition by horizontal transfer (Figure 1, Additional file 2: Table S3). In contrast only 10 *D. solani* 3337-specific regions contain such genes (Table 2 and Additional file 2: Table S2). The most representative example of such an acquisition by horizontal transfer is the large genomic islands located at different positions in both genomes (*D. solani* 3337 GR 24/ *D. dadantii* 3937 GR17 in Figure 1). Interestingly, these islands contain a mixture of conserved and unique genes and present signs of multiple chromosomal rearrangements (Figure 4). Both islands contain the genes encoding the type 4 secretion system, that are flanked by genes related to plasmid mobilization systems, and a region comprising several small genes related to regulators, some of them being related to phage functions. Both islands are also flanked by a conserved integrase and harbor two genes encoding the T6SS-related Hcp and VgrG proteins as well as the related *rhsB* gene.

**Table 2:** **Genomic regions present in *D. solani *** **3337 and absent in *D. dadantii *** **3937**

**Genomic regions**	**Genes ID**	**Presence of horizontal transfer signature**	**Predicted function**	**Presence in other *Dickeya***
GR-1	0295-0300	Yes	Metabolism	
GR-2	0366-0379	Yes	Urea transport and metabolism	*D. chrysanthemi* 1591 (8 genes/13)
GR-3	0946-0954	No	NRPS - PKS	*D. zeae* 586 (5 genes/9)
GR-4	1135-1144	No	-	
GR-5	1151-1160	No	Transport - metabolism	
GR-6	1191-1198	No	Metabolism	
GR-7	1269-1292	No	Transport - metabolism	*D. zeae* 586 (4 genes/20)
GR-8	1634-1640	No	Transport - metabolism	
GR-9	1988-2010	No	PKS - NRPS	*D. paradisiaca* 703 (whole cluster)
GR-10	2094-2099	No	Transport - metabolism	*D. paradisiaca* 703 (whole cluster)
GR-11	2316-2323	Yes	Phage-related	
GR-12	2512-2529	No	Lipid metabolism	*D. zeae* 586 (13 genes/20)
GR-13	2984-3005	Yes	Phage/Tn-related	
GR-14	3131-3143	Yes	Metabolism	
GR-15	3336-3342	No	Transport	
GR-16	3380-3395	Yes	-	
GR-17	3524-3541	Yes	Metabolism	
GR-18	3621-3638	No	Transport - metabolism	*D. zeae* 586 (9 genes/16)
GR-19	4035-4053	No	PKS - NRPS	
GR-20	4101-4110	Yes	Transport - metabolism	
GR-21	4146-4155	No	Metabolism (putative)	
GR-22	4167-4173	No	Metabolism (putative)	
GR-23	4292-4297	Yes	Galactonate transport and metabolism	
GR-24	4305-4355	Yes	*rhsB*- T4SS	
GR-25	4463-4479	No	Metabolism?	

#### *Distinctive metabolic properties between D. solani 3337 and D. dadantii 3937*

**Figure 5:**
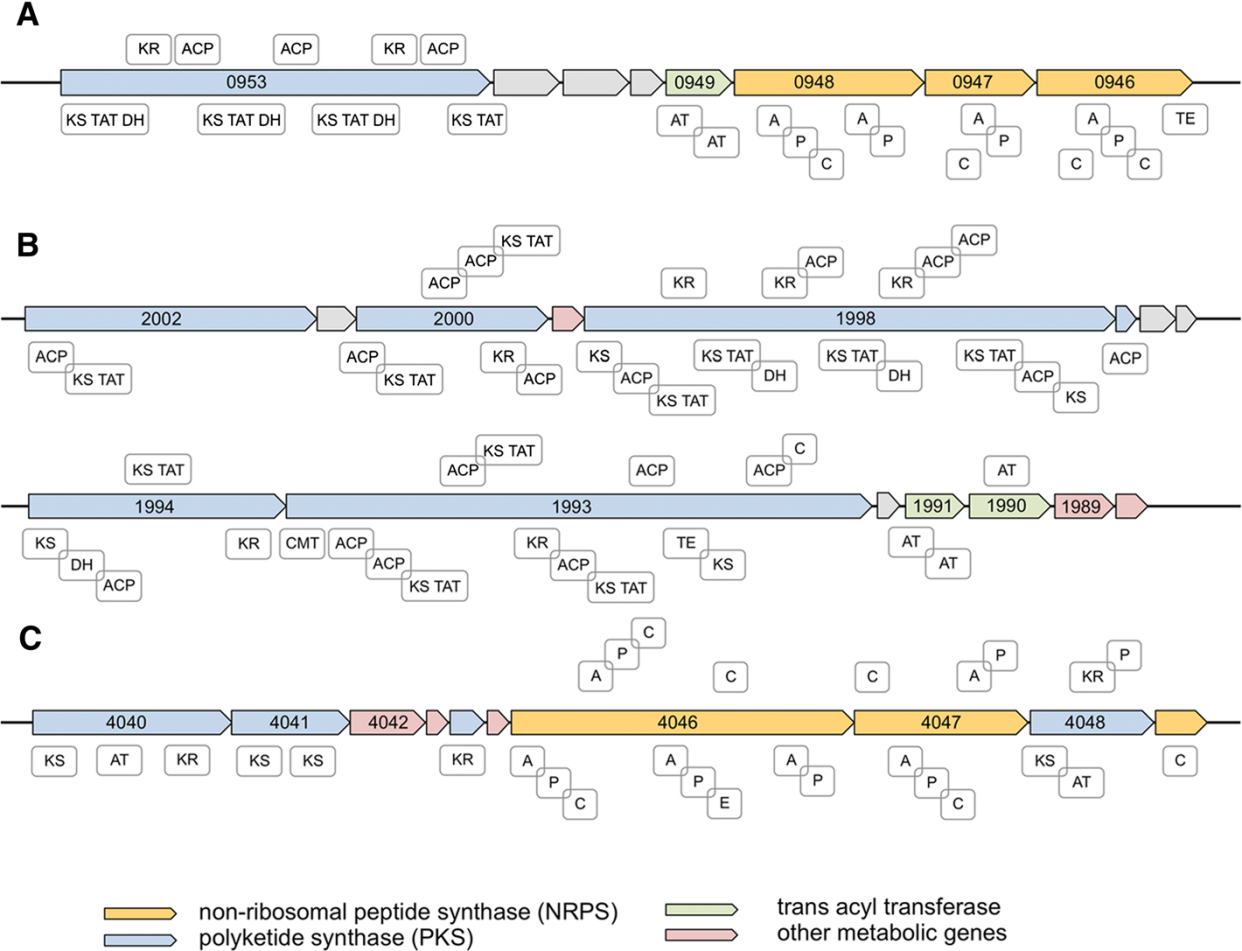
**Representation of the *D. solani *3337 genomic regions regrouping NRPS/PKS genes. A**. GR3 (solani_g3_0946 to solani_g3_0954). **B**. GR9 (solani_g3_1988 to solani_g3_2010) **C**. GR19 (solani_g3_4035-solani_g3_4062) PKS genes are depicted in blue, transacyl transferase genes in green and NRPS genes in yellow. KS: ketoreductase domain, TAT: trans acyltransferase domain, DH: dehydratase domain, KR: keto reductase domain, ACP: acyl carrier domain, AT: acyl transferase domain, A: AMP binding domain, P: peptidyl carrier domain, C: condensation domain, TE: thioesterase domain.

Besides 3 genomic regions consisting of mobile elements and/or phage related genes (GR11, 13, 16, Table 2), 19 *D. solani*-specific genomic regions comprise genes encoding known or putative metabolic pathways (Table 2). Analysis with the AntiSMASH server [44] identified three of these GR as gene clusters that encode complex non ribosomal peptide synthases (NRPS) and polyketide synthases (PKS) as well as associated proteins (GR3, 9, 19, Additional file 2: Table S2, Figure 5). These very large multidomain and multimodular proteins are known to be involved in the biosynthesis of polymers of peptidyl/carbonyl chains that harbor numerous biological activities varying from adaptation to unfavourable environments, competition to other microorganisms or action as virulence factors [45].

Each module allows the addition of one unit of the synthesized polymer. The core module of PKS consists of an acyl carrier protein (ACP) domain for unit loading and a beta-ketosynthase (KS) domain for condensation. For PKS activity, an acyltransferase (AT) domain is also required. This AT activity may be provided either by an AT domain incorporated in each module or by discrete free-standing acyltransferase enzymes in the case of *trans*-AT PKSs. The core module of NRPS consists of an adenylation (A) domain for the selection of one amino acid, a peptidyl carrier protein (PCP) and a condensation domain (C) for catalysing peptide bond formation [45]. Among the *D. solani* genomic regions, GR3 regroups 7 genes related to metabolism among which 3 genes encoding NRPS that regroup 4 synthetic modules, one *trans*-acetyltransferase and a *trans*-AT PKS of 4 modules. This gene cluster appears to be specific to some *Dickeya* since it is found only in *D. zeae* 586 in databases. GR9 regroups 14 metabolic genes among which 7 genes encoding *trans*-AT PKS modules and two *trans*-AT encoding genes. This gene cluster is present in *D. paradisiaca* 703 and in *Serratia odorifera*. GR19 is also a complex gene cluster regrouping a mix of 3 genes encoding NRPS modules and 4 PKS related genes. Beside 11 genes related to production of secondary metabolites, this cluster also encodes one regulatory protein and 2 proteins involved in transport systems. This gene cluster has counterparts in *Serratia* and partly in *Xenorhabdus bovienii*.

The other *D. solani*-specific metabolic regions typically regroup one or more regulators and a few enzymes sometimes accompanied by a transport system. This prompted us to compare the metabolic capacities of *D. dadantii* 3937 and *D. solani* 3337 with BIOLOG plates. The use of 190 compounds as carbon and 95 compounds as nitrogen sources was compared. Among these, 10 compounds as carbon sources and 18 compounds as nitrogen sources were differentially metabolized by *D. dadantii* 3937 and *D. solani* 3337 with a clear enrichment of nitrogen sources that can be metabolized by *D. solani* (Table 3). Interestingly, these differences could be correlated to the presence or absence of metabolic and transport genes. In particular, *D. solani* harbors genes involved in urea metabolism and transport (GR2) that are absent in *D. dadantii* 3937; the assimilation of urea as a nitrogen source by *D. solani* 3337 was validated in a growth assay (Additional file 3: Figure S1). In addition, *D. solani* harbours genes encoding for galactonate assimilation (GR23); the growth of *D. solani* 3337 on galactonate and its γ-lactone derivative as a carbon source was validated in a growth assay (Additional file 3: Figure S1). In contrast, *D. dadantii* 3937 possesses a second citrate metabolic cluster (Dda3937_04561, 04562, 04563 and 00476) that is correlated to an enhanced metabolic ability to use this compound (Table 3). Two of these metabolic genomic regions are also present in *D. zeae* 586 (GR 12 and 18) while GR10 is present in *D. paradisiaca* 703 and GR 2 is present in *D. chrysanthemi* 1591 as well as in *Salmonella* strains (Table 1 and Additional file 2: Table S2).

**Table 3:** **Differential metabolic abilities of *D. dadantii *** **3937 and *D. solani *** **3337**

**PM plates name**	**Compound source name**	**Bacterial strains**	
		***D. dadantii *** **3937**	***D. solani *** **3337**
PM1 MicroPlate™ carbon sources	Formic acid	+^a^	-
	L-Glutamic acid	++	-
	D-Galactonic acid-ﻻ-Lactone	-	++
	Thymidine	+	-
	Uridine	+	-
	L-Glutamine	+	-
	m-TartaricAcid	+	++
	Maltotriose	+	-
	CitricAcid	++	+
PM2A MicroPlate™ carbon sources	SuccinamicAcid	+	-
PM3B MicroPlate™ nitrogen sources	Urea	-	++
	L-GlutamicAcid	-	++
	L-Isoleucine	-	++
	L-Serine	+	++
	L-Tryptophan	+	++
	D-Alanine	-	++
	D-GlutamicAcid	-	+
	Ethanolamine	+	++
	Agmatine	-	++
	Tyramine	+	++
	Xanthine	-	+
	Allantoin	+	++
	Ala-Gln	+	++
	Ala-Gly	-	++
	Ala-His	+	++
	Ala-Leu	+	++
	Ala-Thr	+	++
	Gly-Asn	+	++

^a^, ‘-‘ indicates the absence of metabolism; ‘+’ a weak metabolic capability, and ‘++’ a high metabolic capability (see Methods for details in the measurements).

### The 25 GRs of *D. solani* 3337 are also present in all available draft genomes of *D. solani*

Blast analysis revealed that all the 25 GRs of *D. solani* 3337 are present in the genomes of the *D. solani* strains IPO2222, MK10, MK16 and LMG25865. Similarly, MAUVE analysis shows a complete synteny between these *D. solani* genomes except that the big inversion between *rrs* operons found in *D. solani* 3337 strain as compared to *D. dadantii* 3937 is not present in other *D. solani* strains.

**Figure 6:**
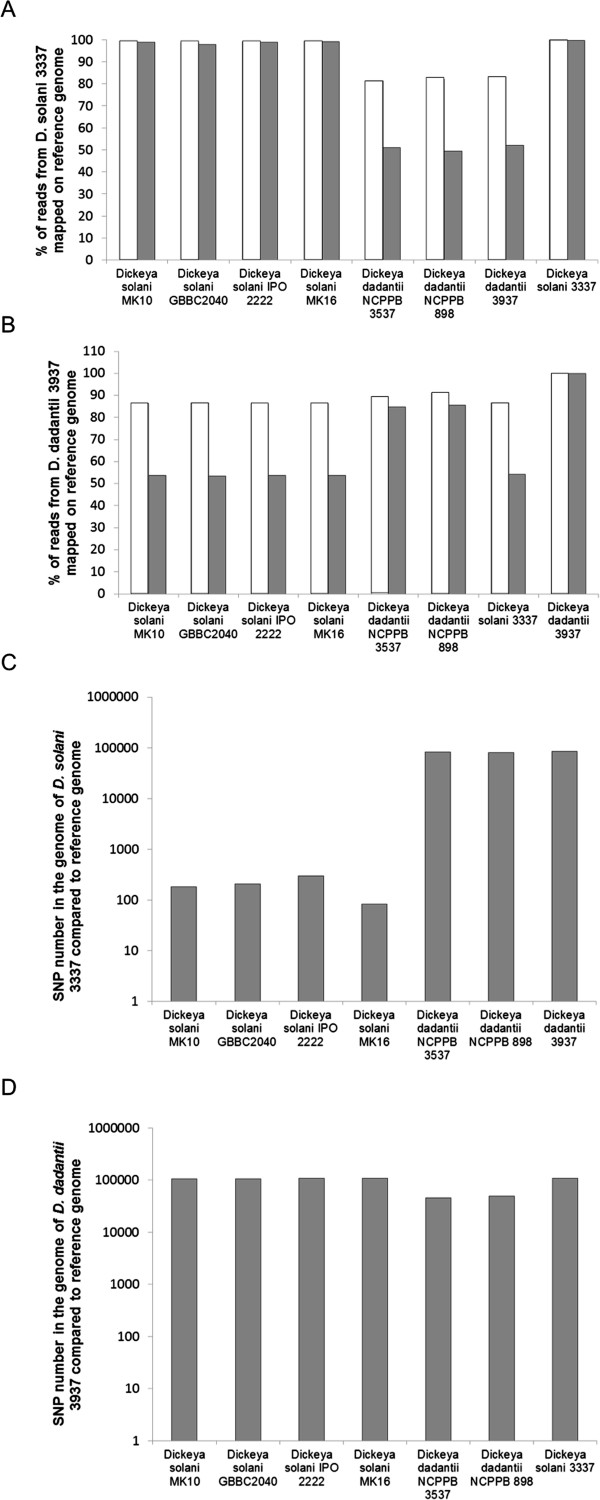
**Genetic diversity in *D. solani *3337 and *D. dadantii *3937. A**. Reads of *D. solani* 3337 mapping on *D. solani* and *D. dadantii* published genomes using mild stringency (white bars) and high stringency (grey bars) parameters. **B**. Simulated reads of *D. solani 393*7 mapping on *D. solani* and *D. dadantii* published genomes using mild stringency (white bars) and high stringency (grey bars) parameters. **C**. Number of SNPs in the genome of *D. solani* 3337 strain as compared with the published *D. solani* and *D. dadantii* genomes. **D**. Number of SNPs in the genome of *D. dadantii* 3937 as compared with the published *D. dadantii* genomes.

Additional genomic comparisons were performed using an *in silico* DNA-DNA hybridization approach. Mapping of the *D. solani* 3337 reads on the *D. solani* and *D. dadantii* present in databases reveals the high similarities of the genomes of the *D. solani* species (Figure 6A). This result reinforces the preliminary data obtained using single-gene sequence data [6]. The same procedure with the generated reads of *D. dadantii* 3937 revealed that the *D. dadantii* strains are more diverse in their genomic content (Figure 6B). These *in silico* genome-hybridization method clearly separated the two species (Figure 6A and B).

The high genome similarity among *D. solani* isolates is confirmed with SNP analysis. Only 200 to 300 SNPs were detected between the genome of *D. solani* 3337 and the other *D. solani* genomes (Figure 6C). In contrast, at least 80,000 SNPs were detected between the *D. dadantii* 3937 genome and the genome of the other tested *D. dadantii* strains (Figure 6D).

## Conclusion

The genome comparison between the potato isolate *D. solani* 3337 and the model *D. dadantii* 3937 only highlights small differences in the arsenal of *Dickeya* virulence factors characterized so far. By contrast, the two strains diverge in their battery of T5SS/T6SS-related toxin-antitoxin systems and harbour distinctive metabolic capacities. In particular, *D. solani* possesses 3 gene clusters regrouping NRPS/PKS genes known to be involved in the biosynthesis of complex secondary metabolites that were shown in other systems to have antibiotic activities or even to be important in virulence against plants [46]. Importantly, the *D. solani*-specific gene clusters are conserved in all draft genomes of *D. solani* that are available until today. These clusters are thus promising for deciphering the molecular mechanisms supporting the recent emergence and lifestyle of the *D. solani* species.

## Abbreviations

NRPS: Non-ribosomal peptide synthase; PKS: Polyketide synthase; bp: Base pairs.

## Competing interests

The authors declare they have no competing interests.

## Authors’ contribution

SM carried out genome sequence assembly and comparative sequence alignment with other *Dickeya* strains, JP and FVG carried out manual annotation and comparative genome annotation analysis, YRE carried out metabolic assays, DF and FVG conceived the study and participate in its design and coordination, all authors participate in manuscript writing. All authors read and approved the final manuscript.

## Supplementary Material

Additional file 1: Table S1 Conservation of T5SS and T6SS-related toxin/antitoxin proteins of *D. solani* 3337 among other *Dickeya.* Click here for file

Additional file 2: Table S2-S3 *D. solani* 3337 (S1) and *D. dadantii* (S2) strain-specific genes. Click here for file

Additional file 3: Figure S1 Metabolic capacities of *D. dadantii* 3937 and *D. solani* 3337. Growth curves of *D.dadantii* 3937 (open squares) and *D.solani* 3337 (closed squares) in the presence of urea as a nitrogen source and galactonate and galactonate γ-lactone as a sole carbon source. Data were collected from triplicates. Click here for file
