# Supplementary material for: Genomic and metabolic comparison with Dickeya dadantii 3937 reveals the emerging Dickeya solani potato pathogen to display distinctive metabolic activities and T5SS/T6SS-related toxin repertoire
Source: BMC Genomics. 2014 Apr 15;15:283. doi: 10.1186/1471-2164-15-283 (PMC4028081; doi:10.1186/1471-2164-15-283)
Supplement: Additional file 1: Table S1 — Conservation of T5SS and T6SS-related toxin/antitoxin proteins of D. solani 3337 among other Dickeya. [file 1471-2164-15-283-S1.DOCX]

| ***D. solani* 3337 gene (ID)** |  | **Identity (%)^1^** | **Cter motif identity (%)^2^** |
| --- | --- | --- | --- |
|  |  |  |  |
| *hecA2* (0501) | *D. dadantii* 3937 | 77 | 29 |
|  | *D. chrysanthemi* 1591 | 66 | 29 |
|  | *D. paradisiaca* 703 | 59 |  |
|  | *D. zeae* 586 | 64 |  |
|  |  |  |  |
| *cdi* (0409) | *D. dadantii* 3937 | 73 | 33 |
|  | *D. chrysanthemi* 1591 | 67 | 32 |
|  | *D. paradisiaca* 703 | 49 |  |
|  | *D. zeae* 586 | 62 |  |
|  |  |  |  |
| *hcpA* (3404) | *D. dadantii* 3937 | 99 |  |
|  | *D. chrysanthemi* 1591 | 98 |  |
|  | *D. paradisiaca* 703 | none |  |
|  | *D. zeae* 586 | 97 |  |
|  |  |  |  |
| *vgrGA* (3403) | *D. dadantii* 3937 | 98 |  |
|  | *D. chrysanthemi* 1591 | 97 |  |
|  | *D. paradisiaca* 703 | none |  |
|  | *D. zeae* 586 | 97 |  |
|  |  |  |  |
| *rhsA* (3401) | *D. dadantii* 3937 | 91 | 100 |
|  | *D. chrysanthemi* 1591 | 93 | 13 |
|  | *D. paradisiaca* 703 | none |  |
|  | *D. zeae* 586 | 90 | 16 |
|  |  |  |  |
| *rhsIA* (3400) | *D. dadantii* 3937 | 100 |  |
|  | *D. chrysanthemi* 1591 | none |  |
|  | *D. paradisiaca* 703 | none |  |
|  | *D. zeae* 586 | none |  |
|  |  |  |  |
| *hcpB* (4345) | *D. dadantii* 3937 | 99 |  |
|  | *D. chrysanthemi* 1591 | 98 |  |
|  | *D. paradisiaca* 703 | none |  |
|  | *D. zeae* 586 | 98 |  |
|  |  |  |  |
| *vgrGB* (4346) | *D. dadantii* 3937 | 88 |  |
|  | *D. chrysanthemi* 1591 | 88 |  |
|  | *D. paradisiaca* 703 | none |  |
|  | *D. zeae* 586 | 89 |  |
|  |  |  |  |
| *rhsB* (4348) | *D. dadantii* 3937 | none* |  |
|  | *D. chrysanthemi* 1591 | none* |  |
|  | *D. paradisiaca* 703 | none |  |
|  | *D. zeae* 586 | none* |  |
|  |  |  |  |
| *rhsIB* (4349) | *D. dadantii* 3937 | none* |  |
|  | *D. chrysanthemi* 1591 | none* |  |
|  | *D. paradisiaca* 703 | none |  |
|  | *D. zeae* 586 | none* |  |
|  |  |  |  |
| *hcpC* (3992) | *D. dadantii* 3937 | 99 |  |
|  | *D. chrysanthemi* 1591 | 98 |  |
|  | *D. paradisiaca* 703 | none |  |
|  | *D. zeae* 586 | 98 |  |
|  |  |  |  |
| *vgrGC* (3993) | *D. dadantii* 3937 | 91 |  |
|  | *D. chrysanthemi* 1591 | 94 |  |
|  | *D. paradisiaca* 703 | none |  |
|  | *D. zeae* 586 | 93 |  |
|  |  |  |  |
| *rhsC* (4000) | *D. dadantii* 3937 | 90 | 24 |
|  | *D. chrysanthemi* 1591 | none* |  |
|  | *D. paradisiaca* 703 | none |  |
|  | *D. zeae* 586 | 85 | 97 |
|  |  |  |  |
| *rhsIC* (4001) | *D. dadantii* 3937 | none* |  |
|  | *D. chrysanthemi* 1591 | none* |  |
|  | *D. paradisiaca* 703 | none |  |
|  | *D. zeae* 586 | 95 |  |

^1^ none: no homologous gene (threshold blastp evalue : 10^-5^). *: gene present but not homologous.

^2^ for toxin encoding genes (*hecA2, cdi, rhsABC*). # / #: when variation is restricted to the Cter motif (Ct toxin domain), number of conserved amino acid in the Cter motif. When no value is indicated, the variation is not restricted to the Cter.
