# Supplementary figures and images for: Genomic and metabolic comparison with Dickeya dadantii 3937 reveals the emerging Dickeya solani potato pathogen to display distinctive metabolic activities and T5SS/T6SS-related toxin repertoire
Source: BMC Genomics. 2014 Apr 15;15:283. doi: 10.1186/1471-2164-15-283 (PMC4028081; doi:10.1186/1471-2164-15-283)

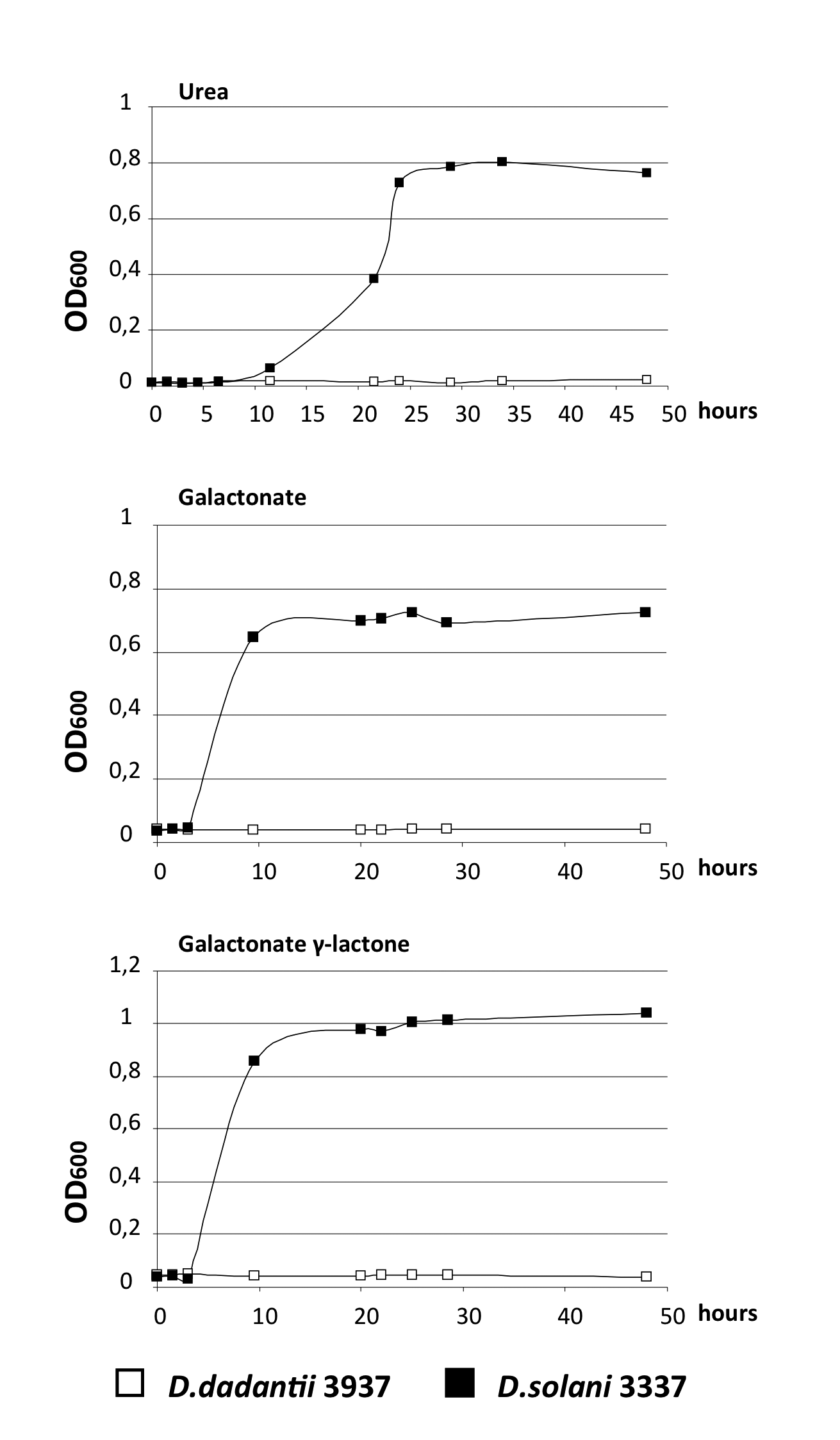

Supplement: Additional file 3: Figure S1 — Metabolic capacities of D. dadantii 3937 and D. solani 3337. Growth curves of D.dadantii 3937 (open squares) and D.solani 3337 (closed squares) in the presence of urea as a nitrogen source and galactonate and galactonate γ-lactone as a sole carbon source. Data were collected from triplicates. [file 1471-2164-15-283-S3.TIFF]
